# Location-Scale Matching for Approximate Quasi-Order Sampling

**DOI:** 10.3389/fpsyg.2019.01163

**Published:** 2019-06-10

**Authors:** Ali Ünlü, Martin Schrepp

**Affiliations:** ^1^TUM School of Education, Technical University of Munich (TUM), Munich, Germany; ^2^SAP SE, Walldorf, Germany

**Keywords:** quasi-order construction, random sampling, representative quasi-order, regression, location-scale matching

## Abstract

Quasi-orders are reflexive and transitive binary relations and have many applications. Examples are the dependencies of mastery among the problems of a psychological test, or methods such as item tree or Boolean analysis that mine for quasi-orders in empirical data. Data mining techniques are typically tested based on simulation studies with unbiased samples of randomly generated quasi-orders. In this paper, we develop techniques for the approximately representative sampling of quasi-orders. Polynomial regression curves are fitted for the mean and standard deviation of quasi-order size as a function of item number. The resulting regression graphs are seen to be quadratic and linear functions, respectively. The extrapolated values for the mean and standard deviation are used to propose two quasi-order sampling techniques. The discrete method matches these location and scale measures with a transformed discrete distribution directly obtained from the sample. The continuous method uses the normal density function with matched expectation and variance. The quasi-orders are constructed according to the biased randomized doubly inductive construction, however they are resampled to become approximately representative following the matched discrete and continuous distributions. In simulations, we investigate the usefulness of these methods. The location-scale matching approach can cope with very large item sets. Close to representative samples of random quasi-orders are constructed for item numbers up to *n* = 400.

## 1. Introduction

We begin with motivating ordered structures and representative quasi-orders, and outline the content and broader scope of the paper.

### 1.1. Ordered Structures

Why are ordered structures such as the quasi-orders important? *Quasi-orders* are reflexive and transitive binary relations (e.g., Davey and Priestley, 2002). They can model, for instance, the dependencies among the items or problems of a psychological test. Dependencies in this context are statements “*The mastery of problem* *y* *of a test* *I* *implies the mastery of problem* *x* *of the test* *I,”* where this statement is modeled as the item pair in relation, *x* ≤ *y*, for a quasi-order ≤ on *I*. A psychological test, equipped with a quasi-order, can be used for the computerized adaptive assessment and training of knowledge. This is realized in *knowledge* or *learning space theory* (KLST) (Doignon and Falmagne, 1985, 1999; Falmagne and Doignon, 2011; Falmagne et al., 2013). The basic idea of KLST is that some pieces of knowledge may imply other pieces of knowledge.

**Figure 1:**
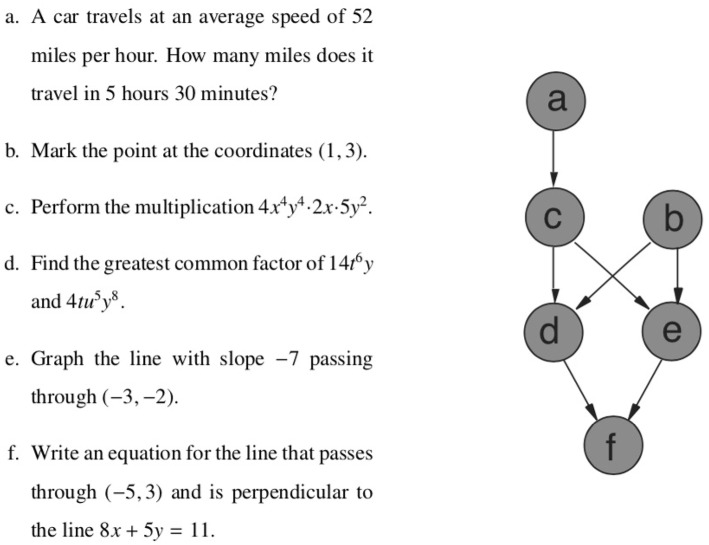
A plausible surmise relation ≤ on the elementary algebra domain {*a*, …, *f*}. For example, the mastery of problem *b* is a prerequisite for the mastery of problem *e* (i.e., *b* ≤ *e*).

An example may be the knowledge domain of elementary algebra. The mastery of an algebra problem *e* [e.g., graph the line with slope −7 passing through (−3, −2)] may imply the mastery of an algebra problem *b* [e.g., mark the point at the coordinates (1, 3)], in particular if the skills required to master problem *e* may also be sufficient to master problem *b*. This is modeled as the item pair *b* ≤ *e* of a quasi-order ≤ on the item set. Because of this interpretation, a quasi-order is also called a *surmise relation* in KLST. This example could consist of six elementary algebra items and may have the following (empirically plausible) quasi-order representation (Figure 1).

Ordered structures also play an important role in other fields, for example in decision theory (Fishburn, 1972; Peterson, 2011), in economics (Varian, 2002) and computer science (Rob and Coronel, 2009), and in sociological questionnaire development (Wiley and Martin, 1999; Martin and Wiley, 2000).

### 1.2. Representative Quasi-Orders

Why are representative random quasi-orders important? In KLST, quasi-orders can be derived by the exploratory data analysis methods of *inductive item tree analysis* (IITA) (van Leeuwe, 1974; Schrepp, 1999, 2003; Sargin and Ünlü, 2009), by querying experts or from postulated theoretical assumptions (e.g., Düntsch and Gediga, 1996; Albert and Lukas, 1999; Cosyn and Thiéry, 2000; Heller, 2004), and based on the connection to skill assignments (e.g., Düntsch and Gediga, 1995; Korossy, 1997; Heller et al., 2013, 2017). In the latter context, of data analysis with skills and knowledge structures, the work by Spoto et al. (2016) is pertinent, which introduces an iterative, data-analytic construction procedure for skill maps. In that work, simulation studies are reported, in which the representative sampling of quasi-orders and, more generally, knowledge structures may be important. Other related problems, where randomly generated representative quasi-orders or knowledge structures may play a role, are the evaluation of general measures to describe the fit of a quasi-ordinal knowledge space to data (Schrepp, 2007), and the effects of errors on the construction of knowledge spaces by querying experts (Schrepp and Held, 1995). In general, any work that uses simulation studies manipulating the quasi-orders (i.e., quasi-ordinal knowledge spaces) or knowledge structures to be reconstructed in those studies, should preferably base their simulations on representative collections of ordered structures.

Our application focus is on IITA. IITA comprises data mining algorithms for the derivation of surmise relations from binary data. The goal of IITA is to reconstruct by data analysis of the observed noisy response patterns, the underlying true dependencies among the items. The input of any IITA analysis is a binary data matrix (subjects represented by rows, items represented by columns), and the output is a quasi-order on the item set. The IITA item hierarchy mining techniques are computational, and typically, evaluated and compared based on extensive simulation studies (Ünlü and Schrepp, 2015, 2016a, 2017). At the basis of these simulation studies is a large set of randomly generated quasi-orders, each of which is posited to represent the true dependencies underlying the simulated data. The simulation studies then aim at assessing the ability of the IITA algorithms to reconstruct these known quasi-orders. To control for this dependency on quasi-order structure necessitates the use of representative random quasi-orders. With representative samples, each quasi-order has the same chance of being included in the simulation study, so you ensure that no interesting quasi-order has been missed.

The *representativeness* of a randomly generated subset, or sample, of quasi-orders on an item set means that each quasi-order on the item set has equal probability of being selected as part of the sample. Ünlü and Schrepp (2015, 2016a, 2017) showed that the use of non-representative samples of quasi-orders led to biased or erroneous conclusions regarding the recovery and coverage qualities of the IITA algorithms in simulation studies. These authors were able to correct the problems induced by non-representative samples with the use of representative random quasi-orders. Thus, it is essential to base any principled simulation study conducted for the reliable, sound comparisons of algorithms used to mine for quasi-orders on unbiased or representative quasi-order samples. In this paper, we introduce two random processes, the normal and discrete location-scale matching methods, for the generation of close to representative samples of random quasi-orders.

### 1.3. Content and Broader Scope

This paper is structured as follows. In section 2, we recapitulate the state-of-the-art techniques available for sampling quasi-orders. In section 3, we discuss polynomial regression analyses for the mean and standard deviation of quasi-order size as a function of item number. (For a set *X*, |*X*| denotes the size of *X*, that is, the number of elements of the set). In section 4, the normal location-scale matching method and the discrete location-scale matching method are introduced. In section 5, we present the simulation results obtained for these methods used to sample quasi-orders. In section 6, we conclude with a summary and suggestions for further research.

Our work can be embedded into, at least, two broader domains. One is in computer science and bioinformatics, the other in computational combinatorial mathematics (see also the first paragraph of section 6.2). In (bio)informatics, the work we present can be seen as a special application in the field of random generation of complex algorithmic structures—for example, Flajolet et al. (1994), Denise et al. (2003), Rodionov and Choo (2003), Duchon et al. (2004), Ponty et al. (2006), and Bassino and Nicaud (2007). This field of research deals with the question of how complex objects encountered in computer science or bioinformatics can be randomly generated, with certain desired properties of their distributions. The objects can be combinatorial structures (e.g., trees) or genomic sequences (i.e., strings of symbols that fulfill specific restrictions). Typical use cases for such generated structures are tests for algorithms that operate on these sorts of structures or the detection of structural information. In (computational) combinatorial mathematics, the problem of sampling quasi-orders can also be put in the broader context of the random generation of complex combinatorial or discrete-mathematical structures—for example, Harary and Palmer (1973), Nijenhuis and Wilf (1978), Dixon and Wilf (1983), Kerber et al. (1990), Brinkmann and McKay (2002, 2005), Pfeiffer (2004), and Roberts and Tesman (2009). There the primary focus is on enumeration or counting the structures (e.g., graphs), rather than uniformly constructing them. However, the works by Dixon and Wilf (1983) and Kerber et al. (1990) studied the uniform generation of unlabeled general graphs.

The above two broader domains cannot be directly applied to the present context, and thus, we have indeed contributed to these domains. In our special case, the mathematical objects considered are the quasi-orders, which should be generated based on the uniform distribution, and the main application of this is a test of algorithms of inductive data analysis, in particular of IITA. For example, we have also contributed to the theory of graphs. Quasi-orders correspond to transitive directed graphs, or finite topologies. It is their counts for different numbers of points that has been studied in the literature (especially of the second broader domain) only, rather than developing feasible algorithms for (close to) uniformly constructing them, even for large numbers of points, as we have done in the present work.

## 2. Existing Sampling Methods for Quasi-Orders

We recapitulate the methods currently available for sampling quasi-orders, categorized into direct, *ad hoc*, and inductive methods.

### 2.1. Direct and *ad hoc* Methods

To construct all possible quasi-orders on an item set and then to draw a random sample from the constructed set is the most direct approach to creating representative random quasi-orders (census-like uniform sampling). Another direct method is to fill the entries of the relational matrix uniformly at random (entry-wise uniform sampling). But obviously, these approaches are only feasible for very small item sets (Schrepp and Ünlü, 2015). Thus, other *ad hoc* random processes (normal and uniform variants) were tried (Schrepp, 1999; Sargin and Ünlü, 2009) to overcome this limitation. However, Ünlü and Schrepp (2015) showed that these *ad hoc* procedures generally do not yield representative quasi-order samples.

### 2.2. Uniform Extension Method

The first, feasible for larger *n* method, called the *uniform extension method* (UEM), was proposed by Schrepp and Ünlü (2015). This method works fine for item numbers up to *n* = 15, but for larger *n*, it too becomes computationally intensive. Therefore, Ünlü and Schrepp (2016b) introduced the more feasible *simple resampling method* (SIRM) and *stratified resampling method* (STRM). With the latter two methods, quasi-order samples were generated up to *n* = 50 items. In the present paper, we propose the new *normal location-scale matching* (NLSM) and *discrete location-scale matching* (DLSM) procedures, which extend the range for feasible item numbers up to *n* = 400.

In the sequel, the UEM, SIRM, and STRM methods will be reviewed. The UEM is inductive. It starts with a representative sample *Q*(*l*) of quasi-orders on a sufficiently small number of items *l*, and constructs from these, by forming random reflexive extensions, a new collection *Q*(*l* + 1) of quasi-orders on *l* + 1 items. More precisely, a relational matrix *r* in *Q*(*l*) is extended with a new randomly filled (*l* + 1)th column and a new randomly filled (*l* + 1)th row, except for the diagonal entry $r_{l+1,l+1}^{\prime }:\;=1$, and leaving intact the original values of *r*. This random reflexive extension is checked for transitivity; if not transitive it is discarded from further analysis, and if transitive it is added to *Q*(*l* + 1). Since random reflexive extensions are used, it can be shown that the sample *Q*(*l* + 1) is representative too (Schrepp and Ünlü, 2015, p. 4, Proposition). The UEM method then constructs from *Q*(*l* + 1), again by taking random reflexive extensions, a collection *Q*(*l* + 2) of representative quasi-orders on *l* + 2 items, and so forth, until the desired item number *n* is achieved.

### 2.3. Randomized Doubly Inductive Construction

We briefly recapitulate the basics of the SIRM and STRM methods, but for details refer the reader to the work by Ünlü and Schrepp (2016b), which introduced these methods meticulously. The SIRM and STRM are defined based on the *randomized doubly inductive construction* (RDIC) (Ünlü and Schrepp, 2016b, section 4.1). The RDIC procedure can be explained as follows. Just like in the UEM, a quasi-order *r*_*n*_ on *n* items is extended to *n* + 1 items by forming a random reflexive extension. Let the random reflexive, but not necessarily transitive, extension of *r*_*n*_ be denoted by $r_{n+1}^{\prime }$. That is, $r_{n+1}^{\prime }$ extends the quasi-order *r*_*n*_ with an extra (*n* + 1)th column and (*n* + 1)th row, the entries of which are randomly filled (except for the diagonal entry, which is 1). In contrast to rejecting the non-transitive extensions as with the UEM method, the RDIC procedure corrects these random reflexive extensions to satisfy transitivity.

**Figure 2:**
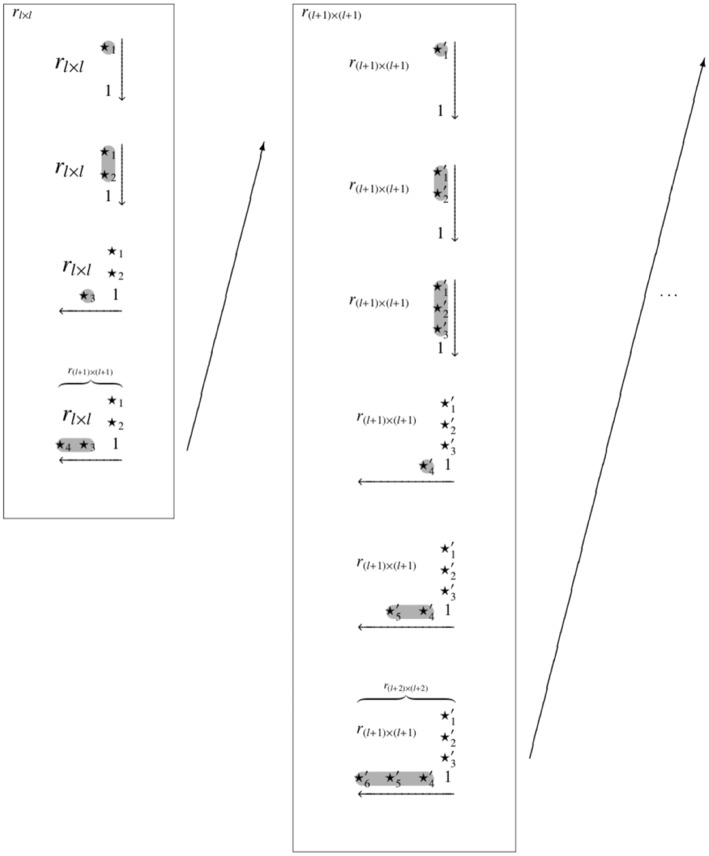
The RDIC procedure exemplified with *l* = 2 items. For one inductive step leading from *l* to *l* + 1 items, and with four and six inductive steps relative to the trace quasi-orders *r*_*l*×*l*_ and *r*_(*l*+1) × (*l*+1)_, respectively. This leads to random reflexive extensions of *r*_*l*×*l*_ on three items and of *r*_(*l*+1) × (*l*+1)_ on four items. The symbols ⋆_*i*_ and ${⋆}_{i}^{\prime }$ denote the added entries that are randomly filled with 0's and 1's.

This is achieved in the following way. Let *r*_1,*n*+1_, …, *r*_*n,n*+1_, the new (*n* + 1)th column, and *r*_*n*+1,*n*_, …, *r*_*n*+1,1_, the new (*n*+1)th row, be the relevant entries of $r_{n+1}^{\prime }$ that need to be corrected if necessary. In this given order and entry by entry (Figure 2), two transitivity conditions (*C*_1_ and *C*_2_) are checked for the (*n* + 1)th column, and for the (*n* + 1)th row three transitivity conditions (*R*_1*a*_, *R*_1*b*_, and *R*_2_) are examined. The transitivity conditions referred to are (Ünlü and Schrepp, 2016b, section 3.1), for *k* = 1, …, *n*:

**Condition C_1_(k), when r_k,n+1_: = 1**. For all *i* ∈ {1, …, *k* − 1}, it holds that *r*_*i,k*_ = 0 or *r*_*i,n* + 1_ = 1.

**Condition C_2_(k), when r_k,n+1_: = 0**. For all *i* ∈ {1, …, *k* − 1}, it holds that *r*_*k,i*_ = 0 or *r*_*i,n* + 1_ = 0.

**Condition R_1a_(k), when r_n+1,k_: = 1**. For all *i* ∈ {1, …, *n*}\{*k*}, it holds that *r*_*i,k*_ = 1 or *r*_*i,n* + 1_ = 0.

**Condition R_1b_(k), when r_n+1,k_: = 1**. For all *i* ∈ {*k* + 1, …, *n*}, it holds that *r*_*k,i*_ = 0 or *r*_*n* + 1,*i*_ = 1.

**Condition R_2_(k), when r_n+1,k_: = 0**. For all *i* ∈ {*k* + 1, …, *n*}, it holds that *r*_*i,k*_ = 0 or *r*_*n* + 1,*i*_ = 0.

Let *x* be a value in the sequence *r*_1,*n*+1_, …, *r*_*n,n*+1_, *r*_*n*+1, *n*_, …, *r*_*n*+1,1_. If the value *x* does not fulfill the respective transitivity condition(s), we replace it with the complementary value 1 − *x*, which then *must* satisfy the transitivity condition(s) (Ünlü and Schrepp, 2016b, p. 8, Proposition 2). On the other hand, if the value *x* is in accordance with the transitivity condition(s), we keep it unchanged. The resulting *corrected* matrix $C\left(r_{n+1}^{\prime }\right)=r_{n+1}$ is the relational matrix of a quasi-order on *n*+1 items, in contrast to the random reflexive extension $r_{n+1}^{\prime }$ of the UEM approach. This quasi-order *r*_*n*+1_ too has the quasi-order *r*_*n*_ as its trace on *n* items.

The overall RDIC sampling procedure, depicted in Figure 2, starts with (the anchoring) a given set *Q*(*l*) of quasi-orders on a sufficiently small item number *l* (e.g., *l* = 2). The quasi-orders in *Q*(*l*) are successively extended, in each step by one more item, forming and correcting random reflexive extensions as described above. This yields new sets of quasi-orders *Q*(*l*+1) for *l*+1 items, *Q*(*l* + 2) for *l* + 2 items, and so forth, until the desired item number *n*, with a sample of quasi-orders *Q*(*n*), is achieved.

With the RDIC procedure, one can generate, quickly and efficiently, samples of quasi-orders on very large item sets. This is an advantage of the RDIC method. However, the RDIC procedure has the disadvantage that the applied corrections are of discrete, combinatorial type. Therefore, the samples constructed according to it are biased (cf. also **Figures 6–8**). For this purpose, bias correction techniques have been proposed (Ünlü and Schrepp, 2016b). Two alternatives for bias correction of the RDIC derived samples are the SIRM and STRM methods. The SIRM and STRM are computationally viable and efficient procedures and provide close to representative quasi-order samples.

### 2.4. Simple Resampling Method

Before we can define the methods SIRM and STRM, we need to introduce the notion of a *biasing position*. Let the entries *r*_1,*n*+1_, …, *r*_*n,n*+1_, *r*_*n*+1,*n*_, …, *r*_*n*+1,1_ of a random reflexive extension be tested in the successive order given according to the RDIC procedure above (Figure 2). Traversed in this order, a position of this sequence is called *biasing* if one, and only one, of the values 0 or 1 satisfies the transitivity condition(s) for this position (Ünlü and Schrepp, 2016b, p. 9, Definition 3).

Then, the following two results hold:

1. According to Ünlü and Schrepp (2016b, p. 8, Part 1 of Proposition 2), for any of the tested entries *r*_1,*n*+1_, …, *r*_*n,n*+1_, *r*_*n*+1,*n*_, …, *r*_*n*+1,1_, at least one of the values 0 or 1 must always satisfy the transitivity condition(s).
2. Let *r*_*n*+1_ be a quasi-order randomly generated from a trace quasi-order *r*_*n*_ according to the RDIC sampling procedure. It can be shown (Ünlü and Schrepp, 2016b, p. 11, Proposition 4) that the probability for sampling *r*_*n*+1_ is $P\left(r_{n+1}\right)=2^{B\left(r_{n+1}\right)}/2^{2n}$, where *B*(*r*_*n*+1_) is the number of the biasing positions among the entries *r*_1,*n*+1_, …, *r*_*n,n*+1_, *r*_*n*+1,*n*_, …, *r*_*n*+1,1_ of *r*_*n*+1_. The weights $2^{-B\left(r_{n+1}\right)}$ and $2^{-B\left(s_{n+1}\right)}$ for two randomly generated quasi-orders *r*_*n*+1_ and *s*_*n*+1_ on *n* + 1 items can be used as the bias correction factors, to adjust for representative or close to representative quasi-order sampling. In this case, we have $P\left(r_{n+1}\right)\cdot 2^{-B\left(r_{n+1}\right)}=1/2^{2n}=P\left(s_{n+1}\right)\cdot 2^{-B\left(s_{n+1}\right)}$.

We define the SIRM method. Suppose a biased multiset (with repetitions) *Q* of quasi-orders has been generated according to the RDIC procedure. To correct for biases, in the SIRM approach, we apply weighted resampling with replacement on this multiset. That is, the weight assigned to an element *r* of *Q* is

(1) $$\begin{array}{l} w_{r}:=\frac{2^{-B\left(r\right)}}{\sum_{r^{\prime }\in Q}2^{-B\left(r^{\prime }\right)}}. \end{array}$$

The values *w*_*r*_ are the probability weights for drawing the quasi-orders *r* ∈ *Q*. The multiset resulting from this weighted resampling with replacement is the SIRM bias-corrected sample. It consists of close to representative random quasi-orders.

### 2.5. Stratified Resampling Method

The STRM method applies bias correction on this multiset *Q* generated according to the RDIC procedure based on stratification, whereby *Q* is partitioned into strata as follows. Let

(2) $$\begin{array}{l} B_{Q}:=\left\{b=B\left(r\right):r\in Q\right\} \end{array}$$

be the set of the unique numbers of the biasing positions of quasi-orders in *Q*. A partition of *Q*, consisting of the strata, is then given by

(3) $$\begin{array}{l} S:=\left\{S_{b}:b\in B_{Q}\right\}, \end{array}$$

with, for *b* ∈ *B*_*Q*_,

(4) $$\begin{array}{l} S_{b}:=\left\{r\in Q:B\left(r\right)=b\right\} \end{array}$$

the submultiset (i.e., stratum) of quasi-orders in *Q* with the same number of biasing positions *b*.

With the STRM method, bias correction of the multiset *Q* is realized by weighted resampling with replacement after stratification, followed by simple random sampling with replacement within the sampled strata. By definition, weighting and resampling the strata $S_{b}\in S$ can be implemented by weighting and resampling the numbers of the biasing positions *b* of *B*_*Q*_. Then, the weight assigned to an element *b* of *B*_*Q*_ is

(5) $$\begin{array}{l} w_{b}:=\frac{|S_{b}|\cdot 2^{-b}}{\sum_{b^{\prime }\in B_{Q}}|S_{b^{\prime }}|\cdot 2^{-b^{\prime }}}, \end{array}$$

with |*S*_*b*_|, *b* ∈ *B*_*Q*_, denoting the size of *S*_*b*_, including repeated membership. The values *w*_*b*_ are the probability weights for drawing *b* ∈ *B*_*Q*_. Let a sample resulting from this weighted resampling with replacement after stratification be denoted by *B*_*S*_.

Simple random sampling with replacement within these obtained strata is realized as follows. Let $B_{S}^{\prime }$ be the set of the unique elements of the multiset *B*_*S*_. For each $b^{*}\in B_{S}^{\prime }$, *m*(b^*^) stands for the number of occurrences of b^*^ in *B*_*S*_. From every stratum $S_{b^{*}}$, $b^{*}\in B_{S}^{\prime }$, a simple random sample with replacement of size *m*(b^*^) is taken. (The discrete uniform distribution on $S_{b^{*}}$, $b^{*}\in B_{S}^{\prime }$ is used.) The resulting bias-corrected sample, and multiset, of close to representative random quasi-orders is the solution of the STRM method.

## 3. Polynomial Regression for Mean and Standard Deviation of Quasi-Order Size

Throughout this paper, “quasi-order size” (number of item pairs in relation) always includes the reflexive item pairs, and the inductive constructions are always anchored/started with the set of all four (labeled) quasi-orders on two items.

### 3.1. Fitting Regression Curves

We perform regression analyses for the mean and standard deviation of quasi-order size as a function of item number. In Table 1, we catalog the quasi-order size means and standard deviations for *n* = 2, …, 20 items. For *n* = 2, …, 6, the true means and true standard deviations were computed in the populations of all possible quasi-orders, which can be constructed for these cases. For *n* = 7, …, 10, the mean and standard deviation averages were taken over ten quasi-order samples each with *N* = 10, 000 quasi-orders simulated with the UEM method. For *n* = 11, …, 20, the averages were computed with 100 samples of *N* = 100, 000 quasi-orders generated according to the SIRM method. The samples generated with the UEM and SIRM methods are (close to) representative in regard to the quasi-order size evaluation criterion (see Schrepp and Ünlü, 2015; Ünlü and Schrepp, 2016b). Thus, the values reported in Table 1 are good estimates of the true quasi-order size means and standard deviations and can be used for regression analyses.

**Table 1:** Quasi-order size means and standard deviations for item numbers *n* = 2, …, 20.

***n***	**Mean**	**Standard deviation**

**Population**		
2	3.000	0.816
3	5.483	1.326
4	8.361	1.855
5	11.612	2.384
6	15.220	2.901
**UEM**		
7	19.149 (0.046)	3.408 (0.032)
8	23.512 (0.094)	3.896 (0.029)
9	28.176 (0.077)	4.407 (0.067)
10	33.174 (0.112)	4.872 (0.053)
**SIRM**		
11	38.519 (0.172)	5.353 (0.091)
12	44.237 (0.202)	5.832 (0.111)
13	50.303 (0.315)	6.323 (0.167)
14	56.730 (0.412)	6.811 (0.203)
15	63.497 (0.544)	7.335 (0.283)
16	70.641 (0.625)	7.826 (0.325)
17	78.065 (0.779)	8.320 (0.349)
18	85.933 (0.896)	8.809 (0.412)
19	94.049 (1.221)	9.331 (0.584)
20	102.497 (1.446)	9.852 (0.691)

**Figure 3:**
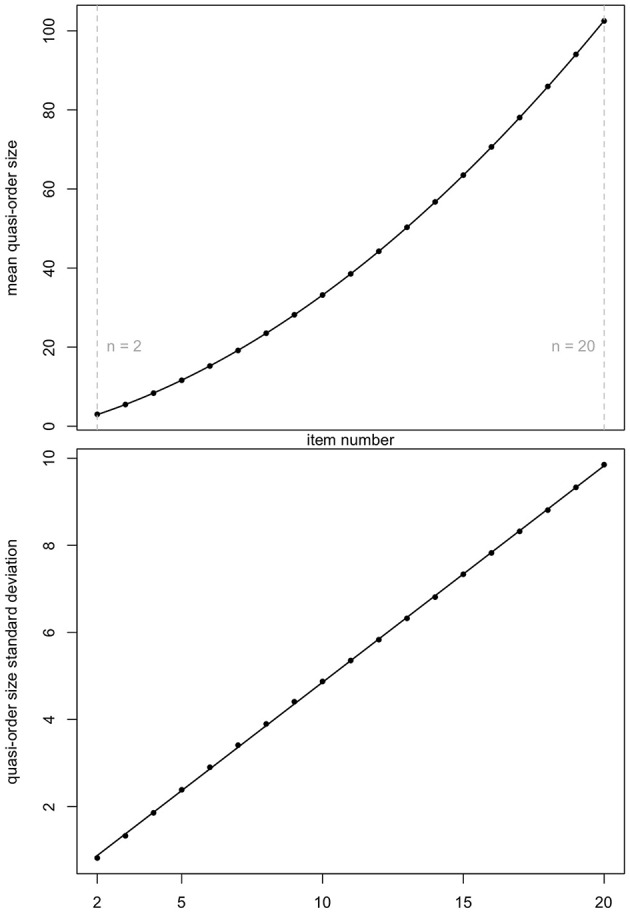
Mean quasi-order size (first row plot) and quasi-order size standard deviation (second row plot) as a function of item numbers *n* = 2, …, 20. The values are taken from Table 1. Fitting polynomial regression through the scatterplots yields quadratic (*R*^2^ = 0.9999989, $R_{\text{adj}}^{2}=0.9999988$) and linear (*R*^2^ = 0.9998774, $R_{\text{adj}}^{2}=0.9998702$) regression lines, respectively. Here, *R*^2^ and $R_{\text{adj}}^{2}$ are the *R*-squared and adjusted *R*-squared, respectively.

*For n = 2, …, 6, the true population values are reported. For n = 7, …, 10, the UEM method was used, and the mean and standard deviation averages were taken over ten quasi-order samples of size N = 10,000. For n = 11,…,20, the averages of the means and standard deviations were computed over 100 samples of N = 100,000 quasi-orders generated with the method SIRM. Standard deviations are in parentheses. The values are plotted in Figure 3*.

Ünlü and Schrepp (2016b) observed that the graph for the mean quasi-order size as a function of the item number was following a quadratic polynomial function. We can see the same trend (top panel of Figure 3). Figure 3 displays the means and standard deviations reported in Table 1.

For both the scatterplots, polynomial regression lines were fitted. In each case, we see a very good fit. In the plots, the *R*-squared (*R*^2^) and adjusted *R*-squared ($R_{\text{adj}}^{2}$) are virtually 1. The resulting quadratic function in the top panel has the equation *q*(*x*) = −1.116 + 1.673*x* + 0.176*x*^2^. The linear function in the bottom panel is *l*(*x*) = −0.121+0.497*x*. Subsequently, we will use these equations to predict the mean quasi-order size and quasi-order size standard deviation for any item number, respectively.

### 3.2. Predictive Analysis

**Figure 4:**
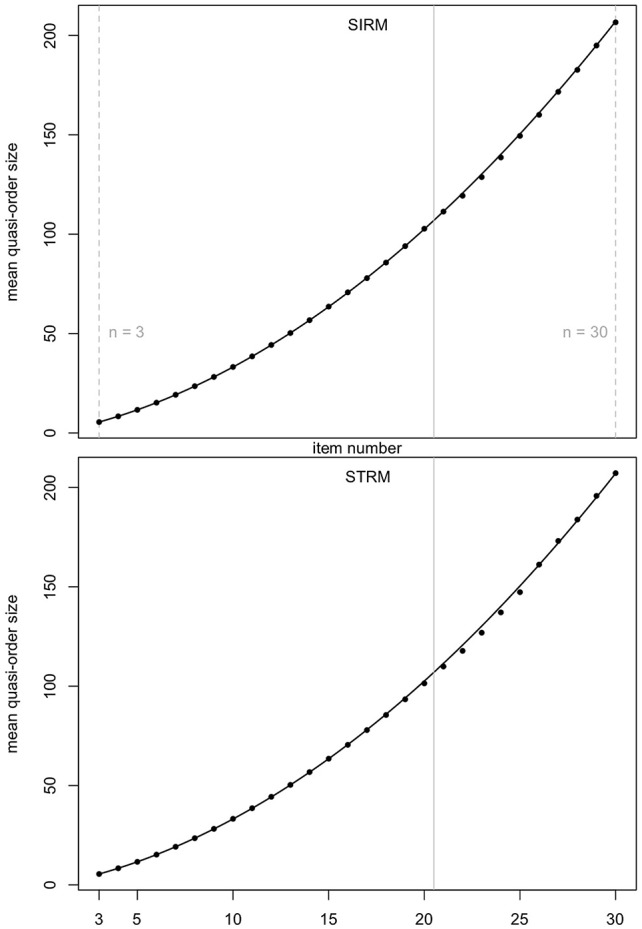
Prediction quality of the quadratic regression line for the mean quasi-order size, for the SIRM and STRM, in first and second row plots, respectively. For any item number *n* = 3, …, 30, one sample of *N* = 500, 000 quasi-orders was simulated based on each of the methods. In every case, the *R*-squared is approximately 1. The vertical solid lines in gray at 20.5 highlight the item range *n* = 21, …, 30.

**Figure 5:**
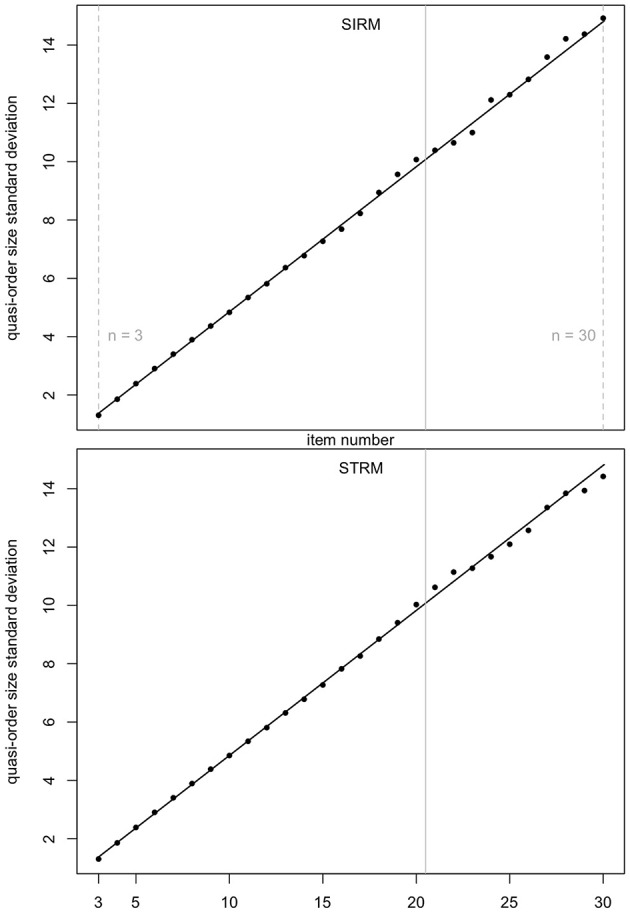
Prediction quality of the linear regression line for the quasi-order size standard deviation, for the SIRM and STRM. For any method, and any item number *n* = 3, …, 30, one sample of *N* = 500, 000 quasi-orders was simulated. In each case, the *R*-squared is approximately 1. The item range *n* = 21, …, 30 is highlighted by the vertical solid lines in gray (at 20.5).

In Figure 4 (mean quasi-order size) and Figure 5 (quasi-order size standard deviation), we test the prediction quality of the regression results with samples generated under the SIRM and STRM methods, for the item numbers *n* = 3, …, 30. Especially the item range *n* = 21, …, 30 (right to the solid gray lines) is informative, since these item numbers were not used in the fitting process. For each item number, one sample of *N* = 500, 000 quasi-orders was simulated under any of the methods SIRM and STRM.

In Figures 4, 5, we see that, even for *n* = 21, …, 30, the fitted regression lines describe the data very well. The computed means and standard deviations all fall close to the fitted lines. For the quadratic function (Figure 4), we obtain *R*^2^ = 0.9999091 (SIRM) and *R*^2^ = 0.9995668 (STRM). For the linear function (Figure 5), the values are *R*^2^ = 0.9985419 (SIRM) and *R*^2^ = 0.9984818 (STRM). Thus, we can assume quadratic or linear relationships between the mean quasi-order size or quasi-order size standard deviation and the item number, respectively.

## 4. Matching Methods

Two matching methods are described, that is to say, the normal and discrete variants. But before we introduce these methods below, let us first summarize the general idea behind them.

What we can observe, in simulations, is that the true (or approximately true) quasi-order size distributions (for larger item numbers) are roughly bell-shaped and symmetric. These distributions have true means, as their locations on the size axis, and true variances, as their scales or spreads. These two parameters (position on the size axis and shape) determine the graph of the distribution. In the case of such a bell-shaped, symmetric distribution as the normal distribution, specifying the mean (location or position) and variance (scale or spread) determines that distribution uniquely. For any item number, the regression predicted mean and standard deviation measures are used to gauge these true location and scale parameters, respectively. This is the meaning of the regression analyses in this paper. Having gauged these true values (by regression), we know where on the size axis the true distribution is located (position) and what its spread (shape) is. Thus, it makes sense (we want to estimate the true distribution), in a next step, to try to match these two summary statistics or properties of the true distribution. This is why we want to match the mean and standard deviation of the predicted regression.

We have to define what we mean by matching. We introduce two definitions, the continuous normal and discrete sample cases (details will be given below). The normal case is defined by using the normal distribution, with the regression predicted true values as its direct (plug-in) mean and variance parameters. As a consequence, this distribution matches the true values, in the sense that these values are reobtained when computing the mean and variance of that distribution. Thus, the corresponding normal probability density function is a good, properly located and spread, proxy for the true quasi-order size distribution. (At this point, note that the true distribution is discrete and approximated by the normal probability density function, which is continuous. This parallels the practice in data analysis when a density function is plotted as an approximation of a histogram.) In particular, we can evaluate this continuous normal proxy (defined on a continuum including the discrete quasi-order sizes) in the discrete sizes directly, to sample the latter. To preserve the approximating normal probability density function curve (including location and scale), we use normalized sampling weights (division by the sum of function values) for the drawn quasi-order sizes (Equation 7).

The discrete case is not so straightforward. We do not have a symmetric discrete distribution with explicit mean (location) and variance (scale) model parameters that could be allocated with the regression predicted true values (as we did for the continuous normal distribution). However, we can operate on the observed quasi-order sizes (the sample) directly. The crucial question then is, whether the observed discrete size distribution can be transformed in a way such that a new discrete distribution results, which has, that is, matches, the regression predicted true values as its computed mean and standard deviation. The answer is yes, and the transformation achieving this is defined by Equation 8. As proved there, this transformation satisfies the pertinent Equation 9, for the matched location, and Equation 10, for the matched scale. Thus, the purpose of this transformation (Equation 8) is to manufacture this properly located and spread new discrete distribution. Albeit their discrete supports differ (the transformed values are not integers in general), the new discrete distribution can be viewed as a good, that is, properly located and spread, proxy for the true discrete distribution. We have two discrete distributions, which coincide approximatively in their location and scale properties. This remains so, if we turn this discrete distribution obtained after transformation into a piecewise linear, continuous function, by linear interpolation, in order to permit the integer-valued observed sizes. Just like in the normal case, the latter continuous function (which is a general function, neither a distribution nor density function) can be viewed as a good proxy for the true discrete distribution. To preserve this curve approximating the true distribution, the sampling weights used for the drawn quasi-order sizes are normalized (Equation 11).

### 4.1. Normal Location-Scale Matching

**Figure 6:**
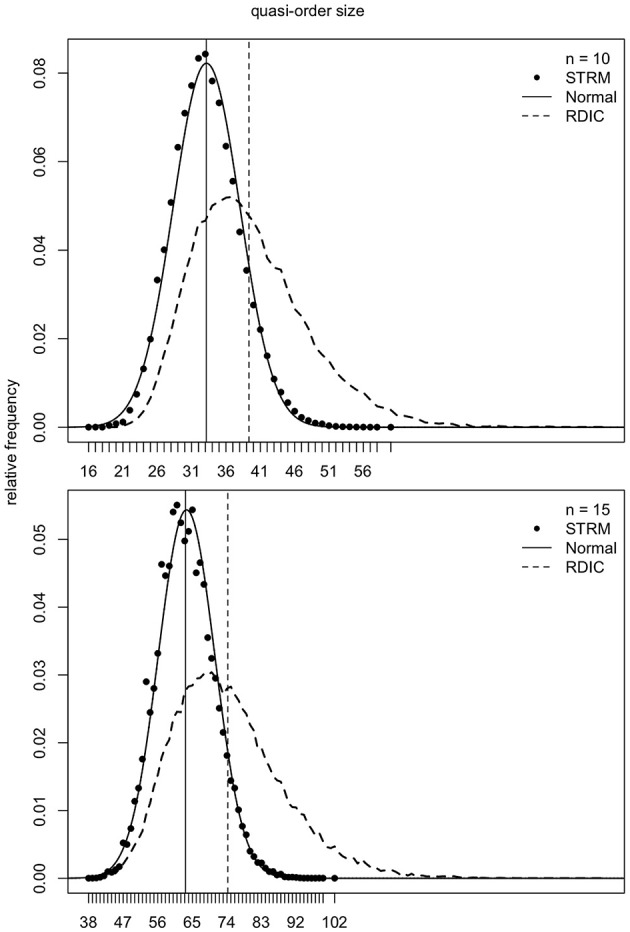
For item numbers *n* = 10 and 15, the relative frequencies (*y*-axes) of the quasi-order sizes (*x*-axes) are shown. For any *n*, the relative frequencies are observed in one sample of *N* = 500, 000 quasi-orders generated under the STRM (filled circles), and in one sample of *N* = 50, 000 quasi-orders constructed with the RDIC (dashed lines). The normal probability density functions with mean and variance parameters set to the values predicted by the fitted quadratic and linear regression functions, respectively, are depicted in solid lines. The vertical solid and dashed lines visualize the sample mean quasi-order sizes for the STRM and RDIC, respectively. The mean quasi-order sizes predicted based on the quadratic regression function under the normal method are virtually the same as for the STRM and represented in vertical solid lines.

**Figure 7:**
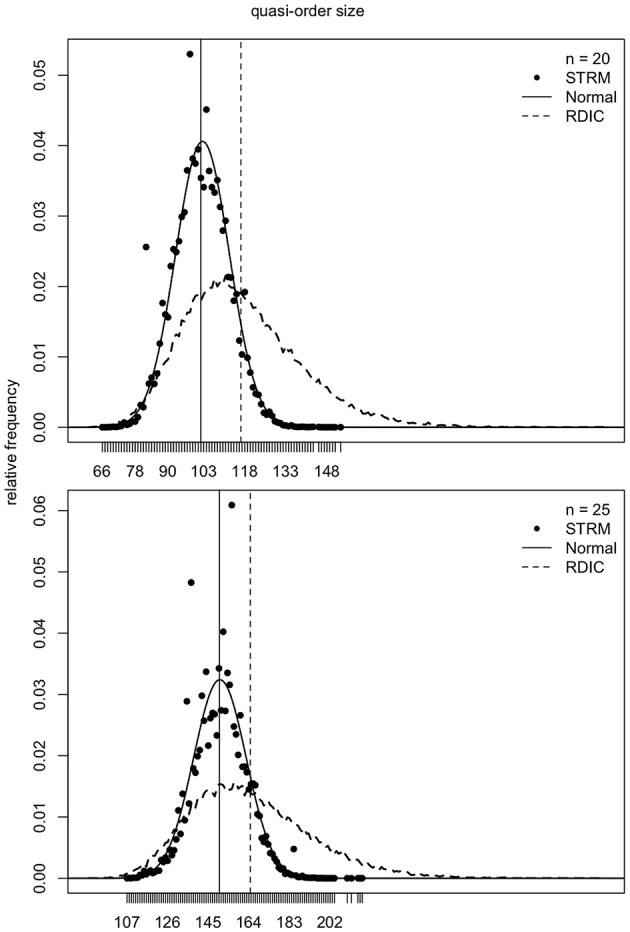
For item numbers *n* = 20 and 25, the relative frequencies of the quasi-order sizes are displayed. For each item number, the relative frequencies are observed in one sample of *N* = 500, 000 quasi-orders under the STRM (filled circles), and in one sample of *N* = 50, 000 quasi-orders with the RDIC (dashed lines). The normal probability density functions with regression predicted mean and variance parameters are fitted through the STRM scatterplots (solid lines). The vertical solid and dashed lines are the sample mean quasi-order sizes for the STRM (≈ normal method) and RDIC, respectively.

**Figure 8:**
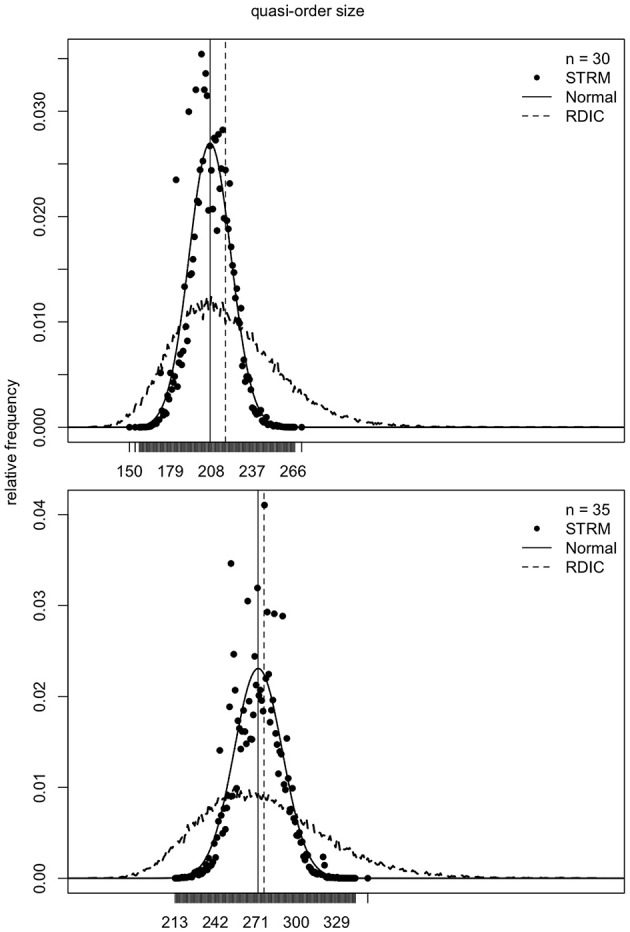
For item numbers *n* = 30 and 35, the quasi-order size relative frequencies are shown. For any *n*, the relative frequencies are observed in one STRM sample of *N* = 500, 000 quasi-orders (filled circles), and in one sample of *N* = 50, 000 quasi-orders constructed with the RDIC (dashed lines). The normal probability density functions with mean and variance parameters set to the values predicted by regression are depicted in solid lines. The sample mean quasi-order sizes are visualized by the vertical solid (STRM and normal method) and dashed (RDIC) lines.

As mentioned in section 2, the samples generated according to the RDIC procedure are biased. This can be seen in Figures 6–8 (cf. also Ünlü and Schrepp, 2016b). In Figures 6–8, the RDIC method is represented by the dashed lines, the STRM method by the filled circles. The normal probability density functions with matched means and standard deviations (see below) are shown in solid lines. The mean quasi-order sizes under the three approaches are depicted as vertical lines, dashed for the RDIC, and solid for the (virtually coincident) STRM and normal method.

In Figures 6–8, we see that, for any item number *n*, the normal probability density function

(6) $$\begin{array}{l} f_{\mu ,\sigma }\left(x\right)=\frac{1}{\sigma \sqrt{2\pi }}\exp\left(-\frac{1}{2}{\left(\frac{x-\mu }{\sigma }\right)}^{2}\right) \end{array}$$

with mean μ: = *q*(*n*) and standard deviation σ: = *l*(*n*) set to the values predicted by the fitted quadratic and linear functions *q*(*n*) = −1.116 + 1.673*n* + 0.176*n*^2^ and *l*(*n*) = −0.121 + 0.497*n*, respectively, provides a good approximation to the reference STRM quasi-order size distribution. We will use these normal densities to sample quasi-orders. A remark is in order. It is not important to have the normal distribution. We could consider any other symmetric, bell-shaped distribution with direct (explicit model parameters) location and scale measures. The normal choice, which satisfies these requirements, is a plausible and convenient one, typically used in statistics. In our application context, the normal distribution fits the data well, as can be seen in the afore mentioned figures.

The normal method, in its formulation of this paper, has at the basis the RDIC generated quasi-order samples. (Instead of the RDIC, other methods for generating the quasi-orders underlying the normal (and also discrete) method could be investigated, as we allude to in the last paragraph of section 6. However, every quasi-order generation procedure, in order to be applicable with the normal (and also discrete) method, has to cope with the problem/remark mentioned in the penultimate paragraph of section 5). Why has the normal method the RDIC at its basis? This method resamples the RDIC constructed quasi-orders to follow the normal probability density function with regression predicted mean and standard deviation parameters (for details see below). The normal method can also be viewed as a variant of bias correction [for other bias correction approaches, see (Ünlü and Schrepp, 2016b)]. That is, we leave the sample biased obtained based on the RDIC, and bias correction is realized through location-scale matching. The latter means shifting combined with stretching or contracting the graph of the quasi-order size distribution implied by the RDIC procedure, to yield the more representative normal density with regression predicted location and scale parameters (cf. also **Figure 11**).

We introduce the *normal location-scale matching* (NLSM) method. We start with a sample *Q*_*N*_(*k*) of quasi-orders on *k* items of size *N*, obtained based on the RDIC. Let *S* = {|*r*_*k*_|:*r*_*k*_ ∈ *Q*_*N*_(*k*)} be the set of the unique quasi-order sizes. Consider the normal density function *f*_μ = *q*(*k*), σ = *l*(*k*)_(*x*) with regression predicted location μ = *q*(*k*) and scale σ = *l*(*k*) parameters. The latter are the extrapolated mean quasi-order size and quasi-order size standard deviation for *k* items. We take a sample of the specified size *N* from the elements of *S*, drawn with replacement. The weight assigned to an element *s*∈*S* is (cf. second and third paragraphs of section 4)

(7) $$\begin{array}{l} w_{s}:=\frac{f_{\mu =q\left(k\right),\sigma =l\left(k\right)}\left(s\right)}{\sum_{s^{\prime }\in S}f_{\mu =q\left(k\right),\sigma =l\left(k\right)}\left(s^{\prime }\right)}. \end{array}$$

The values *w*_*s*_ are the probability weights for drawing the elements of *S*. Let the resulting sample, and multiset, of size *N* be written as *S*′.

What now follows is simple random sampling with replacement. Let *S*″ be the set of the unique elements of the multiset *S*′. For every s^*^ ∈ *S*″, let the number of occurrences of s^*^ in *S*′ be *c*(s^*^). In particular, $\underset{s^{*}\in S^{\prime \prime }}{\sum }c\left(s^{*}\right)=N$. For every s^*^ ∈ *S*″, consider the submultiset $Q_{s^{*}}:=\left\{r_{k}\in Q_{N}\left(k\right):|r_{k}|=s^{*}\right\}$ of quasi-orders in *Q*_*N*_(*k*) with the same quasi-order size s^*^. From each multiset $Q_{s^{*}}$, s^*^ ∈ *S*″, a simple random sample with replacement of size *c*(s^*^) is taken (The discrete uniform distribution on $Q_{s^{*}}$, s^*^ ∈ *S*″ is used. All quasi-orders of $Q_{s^{*}}$, with the same size s^*^ ∈ *S*″, have the same probability of being sampled, $1/|Q_{s^{*}}|$). All quasi-orders obtained in this way are collected in a bias-corrected sample of size *N*, of approximately representative quasi-orders on *k* items. This quasi-order sample constitutes the solution of the NLSM method.

### 4.2. Discrete Location-Scale Matching

There is another approach that can match the mean and standard deviation measures inferred from the polynomial regression. It is the *discrete location-scale matching* (DLSM) method. Again, we start with a multiset *Q*_*N*_(*k*) of quasi-orders on *k* items of size *N*, obtained based on the RDIC (cf. also last paragraph of section 6). Let *S* = {*s* = |*r*_*k*_|:*r*_*k*_ ∈ *Q*_*N*_(*k*)} be the underlying set of the unique quasi-order sizes. For any *s* ∈ *S*, consider the submultiset *Q*_*s*_: = {*r*_*k*_ ∈ *Q*_*N*_(*k*):|*r*_*k*_| = *s*}. These *Q*_*s*_, *s* ∈ *S*, form a partition of the sample *Q*_*N*_(*k*). Let |*Q*_*s*_|, *s* ∈ *S*, stand for the total number of elements, including repeated membership. Let the relative frequencies *f* = (_*f*_*s*_)*s* ∈ *S*_ be *f*_*s*_: = |*Q*_*s*_|/|*Q*_*N*_(*k*)| = |*Q*_*s*_|/*N*, for *s* ∈ *S*. In particular, probabilities are specified, 0 ≤ *f*_*s*_ ≤ 1, *s* ∈ *S*, and $\underset{s\in S}{\sum }f_{s}=1$. We define the sample mean quasi-order size $\bar{\mu }:=\underset{s\in S}{\sum }\left(sf_{s}\right)$ and the sample quasi-order size standard deviation $\bar{\sigma }:=\sqrt{\underset{s\in S}{\sum }\left({\left(s-\bar{\mu }\right)}^{2}f_{s}\right)}$. Let the predicted regression mean and standard deviation be μ_*t*_: = *q*(*k*) and σ_*t*_: = *l*(*k*), where *q* and *l* are the fitted quadratic and linear functions, respectively.

We use the transformed values (cf. second and fourth paragraphs of section 4)

(8) $$\begin{array}{l} t\left(s\right):=\sigma_{t}\frac{s-\bar{\mu }}{\bar{\sigma }}+\mu_{t},\text{for any}\;s\in S. \end{array}$$

To each *t*(*s*) is assigned the probability *f*_*s*_, for *s* ∈ *S*. We obtain the set of points {(*t*(*s*), *f*_*s*_):*s* ∈ *S*}. We can show that

(9) $$\begin{array}{l} \text{(a)}\;\underset{s\in S}{\sum }\left(t\left(s\right)f_{s}\right)=\mu_{t}, \end{array}$$

and

(10) $$\begin{array}{l} \text{(b)}\;\sqrt{\underset{s\in S}{\sum }\left({\left(t\left(s\right)-\mu_{t}\right)}^{2}f_{s}\right)}=\sigma_{t}. \end{array}$$

That is, the discrete distribution {(*t*(*s*), *f*_*s*_):*s* ∈ *S*} has the matched mean (location) μ_*t*_ and standard deviation (scale) σ_*t*_.

Re (a), we have:

$$\begin{array}{l} \underset{s\in S}{\sum }\left(t\left(s\right)f_{s}\right)=\underset{s\in S}{\sum }\left(\left(\sigma_{t}\frac{s-\bar{\mu }}{\bar{\sigma }}+\mu_{t}\right)f_{s}\right)\\ \;=\frac{\sigma_{t}}{\bar{\sigma }}\underset{s\in S}{\sum }\left(\left(s-\bar{\mu }\right)f_{s}\right)+\mu_{t}\underset{s\in S}{\sum }f_{s}\\ \;=\frac{\sigma_{t}}{\bar{\sigma }}\left(\underset{s\in S}{\sum }\left(sf_{s}\right)-\bar{\mu }\underset{s\in S}{\sum }f_{s}\right)+\mu_{t}\\ \;=\mu_{t}. \end{array}$$

Re (b), it holds:

$$\begin{array}{l} \sqrt{\underset{s\in S}{\sum }\left({\left(t\left(s\right)-\mu_{t}\right)}^{2}f_{s}\right)}=\sqrt{\underset{s\in S}{\sum }\left({\left(\sigma_{t}\frac{s-\bar{\mu }}{\bar{\sigma }}+\mu_{t}-\mu_{t}\right)}^{2}f_{s}\right)}\\ \;=\frac{\sigma_{t}}{\bar{\sigma }}\sqrt{\underset{s\in S}{\sum }\left({\left(s-\bar{\mu }\right)}^{2}f_{s}\right)}\\ \;=\sigma_{t}. \end{array}$$

Thus, we have transformed the initial discrete distribution {(*s, f*_*s*_):*s* ∈ *S*} to the discrete distribution {(*t*(*s*), *f*_*s*_):*s* ∈ *S*}, such that the mean and standard deviation match the regression predicted values. However, we cannot directly use this distribution to sample quasi-orders. In general, the transformed *t*(*s*), *s* ∈ *S*, are not integer-valued. To include the integer values *s* ∈ *S*, we perform linear interpolation for the given set of points {(*t*(*s*), *f*_*s*_):*s* ∈ *S*}. The resulting piecewise linear function determined by these points is denoted by *L*. The function *L* has as its domain the interval *I*: = [min{*t*(*s*):*s* ∈ *S*}, max{*t*(*s*):*s* ∈ *S*}]. That is, we do not use interpolation outside the interval *I*.

We can use this function *L* to sample quasi-orders. We take a sample of size *N* from the elements of *S* ∩ *I*, drawn with replacement. The sampling weight for any *s* ∈ *S* ∩ *I* is

(11) $$\begin{array}{l} \frac{L\left(s\right)}{\sum_{s^{\prime }\in S\cap I}L\left(s^{\prime }\right)}. \end{array}$$

Following the line of reasoning for the NLSM method above, we can infer in an analogous manner that a bias-corrected sample of size *N* of approximately representative quasi-orders on *k* items can be constructed. This quasi-order sample defines the DLSM method.

## 5. Simulations

As the evaluation criterion used to assess representativeness, we will primarily focus on the size of a quasi-order. Quasi-order size distributions will be compared for the SIRM, NLSM, DLSM, and the pointwise average taken over both the NLSM and DLSM distribution functions. We will catalog the mean as a location measure and the standard deviation as a scale parameter for the regression results, the SIRM, NLSM, DLSM, and their average. In addition, other criteria such as the *height* (i.e., size of a longest chain) and number of *maximal elements* (i.e., elements not in relation to any other element) will be reported. We will simulate large quasi-orders for item numbers up to *n* = 400. The computations were performed in R (The R Core Team, 2018, www.R-project.org) on an iMac 3.4 GHz Intel Core i7, with memory 32 GB 1,600 MHz DDR3.

### 5.1. Height and Number of Maximal Elements

To begin with, it is important to note that the methods NLSM and DLSM are only approximate, but flexible, and they are defined essentially based on the size criterion. That is, by matching the location and scale measures of a quasi-order size distribution, these methods are designed to approximate that size distribution. However, we can also compare the NLSM and DLSM methods with respect to other evaluation criteria used to assess representativeness.

In Table 2, we report the height and number of maximal elements for item numbers *n* = 3, …, 8. For *n* = 3, …, 6, the populations of all quasi-orders are known, and thus, the true means are presented (“True”). For *n* = 7, 8, the UEM was used as the reference, and the averaged mean values were computed based on ten samples each of *N* = 1, 000 quasi-orders. The mean (over 1, 000 quasi-orders) was computed in each of the samples and averaged over the (ten) samples. For the NLSM and DSLM, we used 100 quasi-order samples each of size *N* = 1, 000.

**Table 2:** Averaged mean height and averaged mean number of maximal elements for the NLSM and DLSM, with the true arithmetic means (“True”) and the UEM as the references, for item numbers *n* = 3, …, 8.

**Criterion**	**NLSM**	**DLSM**	**True**	**Δ_NLSM_**	**Δ_DLSM_**
*n* = 3					
Height	2.412 (0.020)	2.397 (0.019)	2.414	−0.002	−0.017
Maximal	1.245 (0.024)	1.243 (0.030)	1.241	0.004	0.002
*n* = 4					
Height	2.911 (0.031)	2.937 (0.032)	2.904	0.007	0.033
Maximal	1.348 (0.032)	1.290 (0.035)	1.465	−0.117	−0.175
*n* = 5					
Height	3.321 (0.027)	3.358 (0.035)	3.310	0.011	0.048
Maximal	1.560 (0.034)	1.522 (0.035)	1.684	−0.124	−0.162
*n* = 6					
Height	3.658 (0.033)	3.678 (0.040)	3.625	0.033	0.053
Maximal	1.763 (0.035)	1.757 (0.033)	1.899	−0.136	−0.142
**Criterion**	**NLSM**	**DLSM**	**UEM**	Δ_NLSM_	Δ_DLSM_
*n* = 7					
Height	3.968 (0.033)	3.969 (0.035)	3.897 (0.064)	0.071	0.072
Maximal	1.973 (0.037)	1.974 (0.041)	2.094 (0.050)	−0.121	−0.120
*n* = 8					
Height	4.237 (0.032)	4.241 (0.046)	4.103 (0.039)	0.134	0.138
Maximal	2.179 (0.039)	2.183 (0.046)	2.281 (0.068)	−0.102	−0.098

*The true mean values computed in the populations of all possible quasi-orders (n = 3,…,6) are collected in the column “True”. For NLSM and DLSM (n = 3,…,8), the averaged mean values were calculated using 100 quasi-order samples of size N = 1,000. For UEM as the reference (n = 7,8), we used ten samples each with N = 1,000 simulated quasi-orders. The differences “NLSM − True” and “NLSM − UEM” are denoted with Δ_NLSM_, and Δ_DLSM_ stands for the differences “DLSM − True” and “DLSM − UEM”. Standard deviations are in parentheses*.

In Table 2, we can see that the methods NLSM and DLSM are only approximate. There are deviations, however the criterion values obtained for the NLSM and DLSM lie not far away from the “True” or UEM reference values. Similar results can also be obtained for other quasi-order properties, for example the width or number of minimal elements.

### 5.2. Size

**Figure 9:**
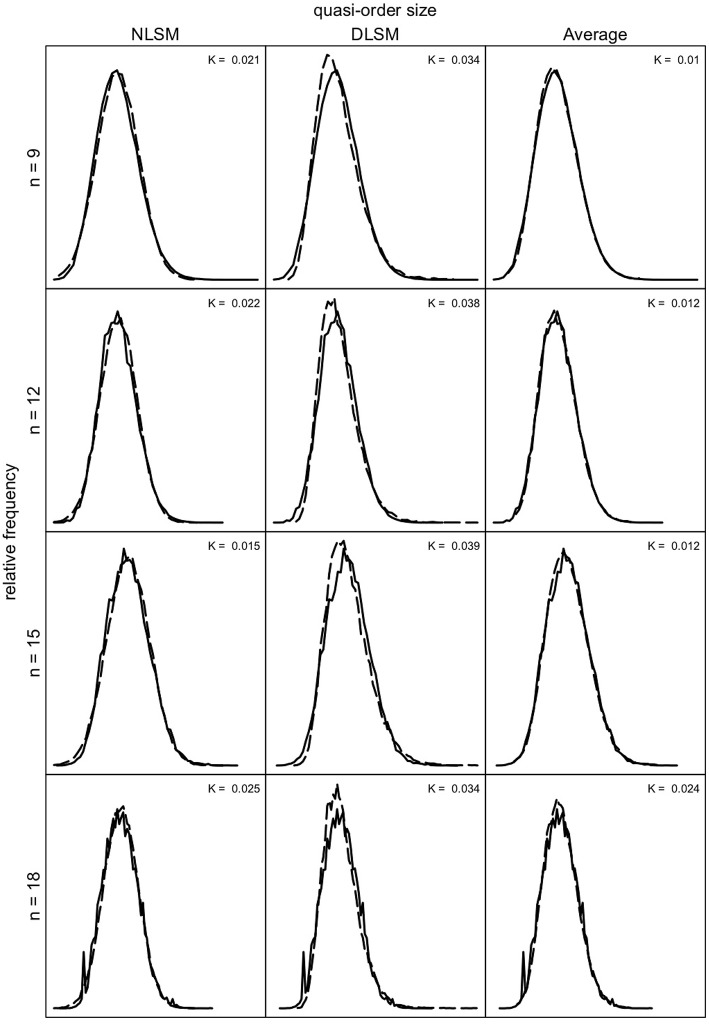
Quasi-order size distributions for the SIRM as the reference (solid lines), with NLSM (dashed lines) as the first column, DLSM (dashed lines) as the second column, and the average of the NLSM and DLSM (“Average”) (dashed lines) as the third column, for item numbers (rows) *n* = 9, 12, 15, and 18. In each row (for each *n*), the same quasi-order size distribution under the SIRM is plotted three times, for the NLSM, DLSM, and “Average” columns. The values *K* stand for the Kolmogorov distances between the NLSM, DLSM, or their “Average” distributions and the SIRM distributions. For the SIRM, we used one sample of *N* = 500, 000 quasi-orders, for any *n*. For the NLSM and DLSM, for any *n*, each method was based on one quasi-order sample of size *N* = 75, 000.

**Figure 10:**
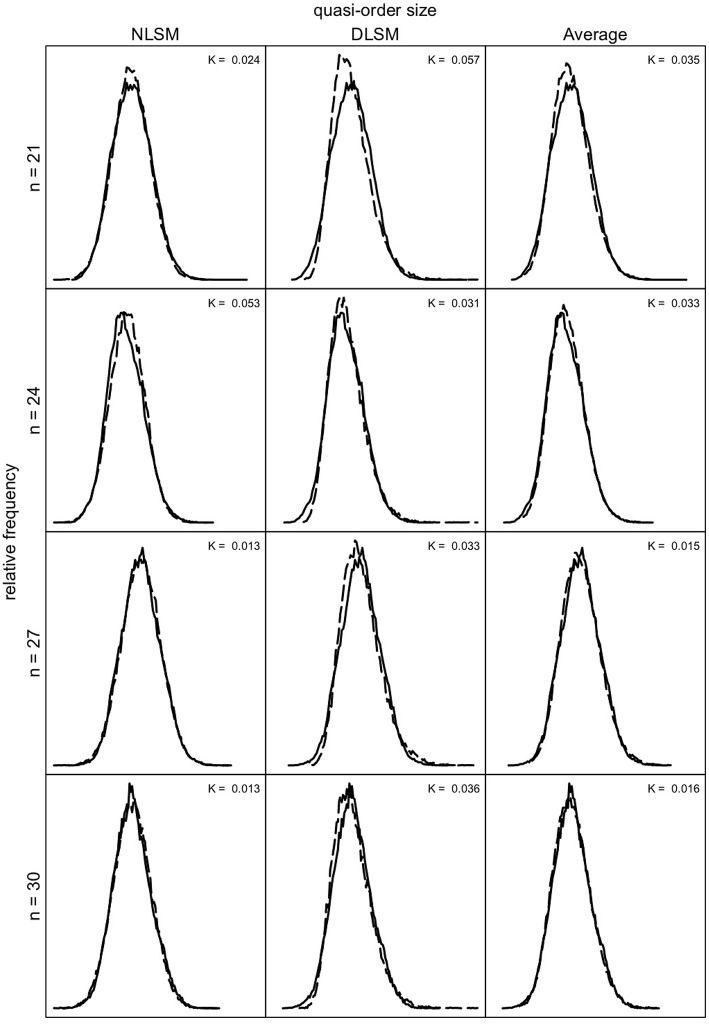
Quasi-order size distributions for the NLSM, DLSM, and their “Average”, each in dashed lines, with the SIRM as the reference in solid lines, for item numbers *n* = 21, 24, 27, and 30. In each row, the same SIRM distribution is plotted three times (in the three columns). The values *K* are the Kolmogorov distances between the NLSM, DLSM, or the “Average” distributions and the SIRM distributions. For any *n*, one sample of size *N* = 500, 000 quasi-orders was used for the SIRM, with duplicates being removed. For the NLSM and DLSM, for any *n*, each used one quasi-order sample of size *N* = 75, 000.

We can investigate how well the quasi-order size distributions obtained for the NLSM and DLSM approximate the size distributions under the SIRM as a representative reference. In Figures 9, 10, we compare these distributions for the item numbers *n* = 9, 12, 15, and 18 and *n* = 21, 24, 27, and 30, respectively. Under the SIRM (solid lines), for any *n*, we simulated *N* = 500, 000 quasi-orders. For the NLSM and DLSM (dashed lines), for any *n*, we used *N* = 75, 000 quasi-orders. The pointwise average function of the two distribution functions under the NLSM and DLSM is denoted by “Average” (dashed lines) in Figures 9, 10. The “Average” will be seen to be the best performing variant in terms of the size criterion, for smaller item numbers. In Figures 9, 10, the Kolmogorov distances *K* of the NLSM, DLSM, and “Average” distributions with respect to the SIRM distributions were also computed.

As can be seen in Figures 9, 10, the NLSM and “Average” provide better approximations to the representative SIRM distributions compared to the DLSM. The distributions for the DLSM are good approximations too, however they are slightly shifted to the left. In Figure 9, the Kolmogorov distances are smallest for the “Average,” followed by the NLSM, with worst results obtained for the DLSM. We see that, for the specific item range *n* = 9, 12, 15, and 18, the “Average” slightly outperforms the NLSM. Hence, if this is the range of interest, the method of choice could be the “Average.” For larger item numbers, the NLSM is the best choice. Overall, across the item numbers *n* = 9, 12, 15, 18, 21, 24, 27, and 30, the NLSM, DLSM, and “Average” provide approximate distributions close to the representative SIRM. In addition, in Figures 9, 10, we can see that the mean and standard deviation values obtained under each of the methods are comparable; they are close to each other. This can also be seen in Table 3.

**Table 3:** Location (μ) and scale (σ) measures for the regression solution, SIRM, NLSM, DLSM, and the average of the NLSM and DLSM, for item numbers *n* = 7, …, 30.

***n***	**μ_*t*_**	**σ_*t*_**	**μ_SIRM_**	**σ_SIRM_**	**μ_NLSM_**	**σ_NLSM_**	**μ_DLSM_**	**σ_DLSM_**	**μ_Average_**	**σ_Average_**

7	19.198	3.361	19.203	3.401	19.228	3.327	19.334	3.641	19.194	3.397
8	23.505	3.858	23.524	3.895	23.525	3.803	23.545	3.972	23.421	3.771
9	28.162	4.356	28.177	4.365	28.206	4.323	28.206	4.422	28.133	4.290
10	33.171	4.853	33.209	4.834	33.209	4.821	33.202	4.920	33.135	4.799
11	38.531	5.350	38.522	5.342	38.525	5.314	38.527	5.380	38.419	5.257
12	44.242	5.848	44.259	5.813	44.238	5.844	44.257	5.917	44.115	5.755
13	50.304	6.345	50.289	6.365	50.319	6.305	50.330	6.395	50.186	6.228
14	56.717	6.843	56.734	6.776	56.744	6.815	56.726	6.845	56.553	6.704
15	63.482	7.340	63.555	7.270	63.493	7.322	63.494	7.398	63.320	7.219
16	70.597	7.837	70.734	7.687	70.584	7.791	70.625	7.861	70.449	7.711
17	78.064	8.335	77.878	8.226	78.094	8.344	78.086	8.363	77.869	8.215
18	85.882	8.832	85.718	8.943	85.888	8.809	85.905	8.874	85.750	8.733
19	94.050	9.330	94.747	9.473	94.095	9.345	94.086	9.494	93.815	9.225
20	102.570	9.827	103.114	10.137	102.543	9.859	102.619	9.940	102.180	9.643
21	111.442	10.324	111.835	10.701	111.429	10.340	111.478	10.385	111.083	10.119
22	120.664	10.822	120.987	11.626	120.684	10.777	120.727	10.940	120.537	10.727
23	130.237	11.319	129.842	12.021	130.166	11.292	130.226	11.504	129.788	11.147
24	140.162	11.817	139.449	12.128	140.149	11.784	140.139	11.908	139.855	11.672
25	150.437	12.314	149.895	12.394	150.462	12.260	150.573	12.624	150.070	12.155
26	161.064	12.812	160.905	12.713	161.125	12.831	161.124	13.045	160.737	12.676
27	172.042	13.309	171.870	13.180	172.027	13.317	172.114	13.375	171.484	13.076
28	183.371	13.806	183.631	14.075	183.463	13.753	183.470	13.899	182.941	13.577
29	195.051	14.304	194.889	14.594	195.017	14.292	195.005	14.677	194.430	14.121
30	207.082	14.801	207.239	14.795	207.071	14.805	207.097	15.042	206.581	14.658

*The predicted regression mean and standard deviation are μ_t_ = q(n) and σ_t_ = l(n), with q and l the quadratic and linear functions, respectively. For any n, the mean and standard deviation of the quasi-order sizes computed in one sample of N = 500,000 quasi-orders generated under the SIRM are denoted with μ_SIRM_ and σ_SIRM_, respectively. For n≥ 19, for the SIRM, duplicates were excluded. For any n, for the NLSM and DLSM, one sample consisting of N = 75,000 quasi-orders was simulated under each of the methods. The mean and standard deviation of the quasi-order sizes computed in these samples are denoted with μ_NLSM_ and σ_NLSM_, and μ_DLSM_ and σ_DLSM_, respectively. The respective values under the pointwise average function of the NLSM and DLSM distributions are read as μ_Average_ and σ_Average_*.

Table 3 summarizes the means and standard deviations of the quasi-order sizes computed for the regression solution, SIRM (for any *n*, *N* = 500, 000 simulated quasi-orders), NLSM, DLSM, and the average of the NLSM and DLSM (for any *n*, each with *N* = 75, 000 drawn quasi-orders), for item numbers *n* = 7, …, 30.

In Table 3, we can see that the location and scale measures are very close to each other. Whereas the mean is virtually the same across all methods, the standard deviation has a more larger variation. But the standard deviation values are approximately the same.

### 5.3. Large Quasi-Orders

**Figure 11:**
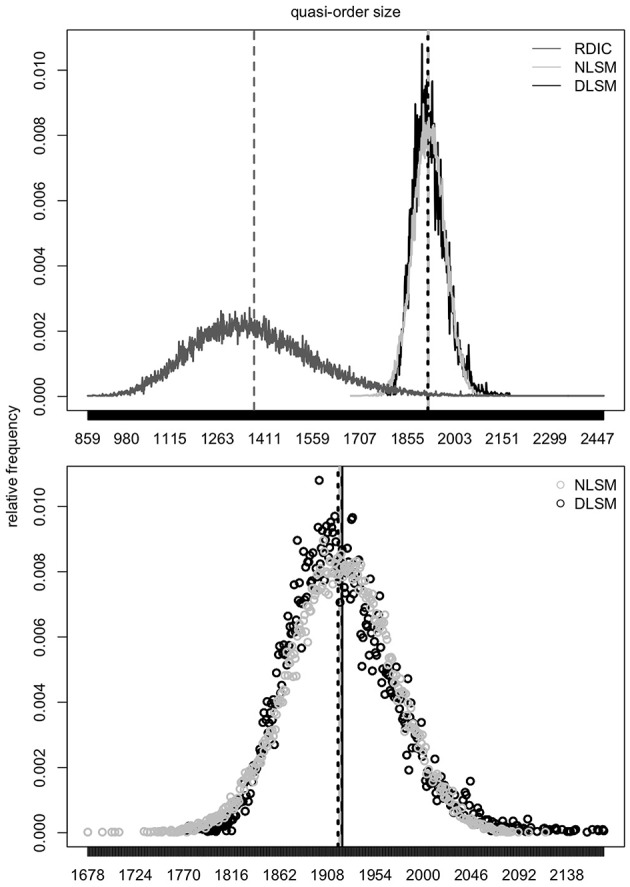
In the first row plot, for *n* = 100, distributions of the quasi-order sizes for one sample of *N* = 50, 000 quasi-orders generated under each of the methods NLSM (gray), DLSM (black), and RDIC (dark gray) are plotted. The vertical dashed lines in gray (NLSM), dark gray (RDIC), and in black (DLSM) represent mean quasi-order sizes. In the second row plot, for *n* = 100, the corresponding scatterplots of the quasi-order sizes for the NLSM (unfilled gray circles) and DLSM (unfilled black circles) are shown. The vertical dashed lines in gray and black portray the mean quasi-order sizes obtained under the NLSM and DLSM, respectively. The vertical solid black line is the quasi-order size mean value predicted by regression.

Based on location-scale matching we can simulate large quasi-orders for item numbers up to *n* = 400. In Figure 11, for *n* = 100, samples of *N* = 50, 000 quasi-orders generated under each of the methods NLSM, DLSM, and RDIC are considered. The quasi-order sizes observed in these samples are plotted.

According to the top panel plot, we can gauge the effect of location-scale matching. The RDIC (solid, in dark gray) sample implies a mean 1, 376.941 (dashed, in dark gray) and standard deviation 189.310. The respective values predicted by regression are 1, 921.808 and 49.620. The RDIC graph is contracted and shifted with the scale and location parameters, respectively, to become the NLSM or DLSM graphs. The NLSM (solid, in gray) yields a mean 1, 919.630 (dashed, in gray) and standard deviation 48.180. The values for the DLSM (solid, in black) are 1, 918.143 (dashed, in black) and 48.742, respectively. The bottom panel scatterplots zoom in on the points of the size distributions and show roughly bell-shaped distribution forms.

**Figure 12:**
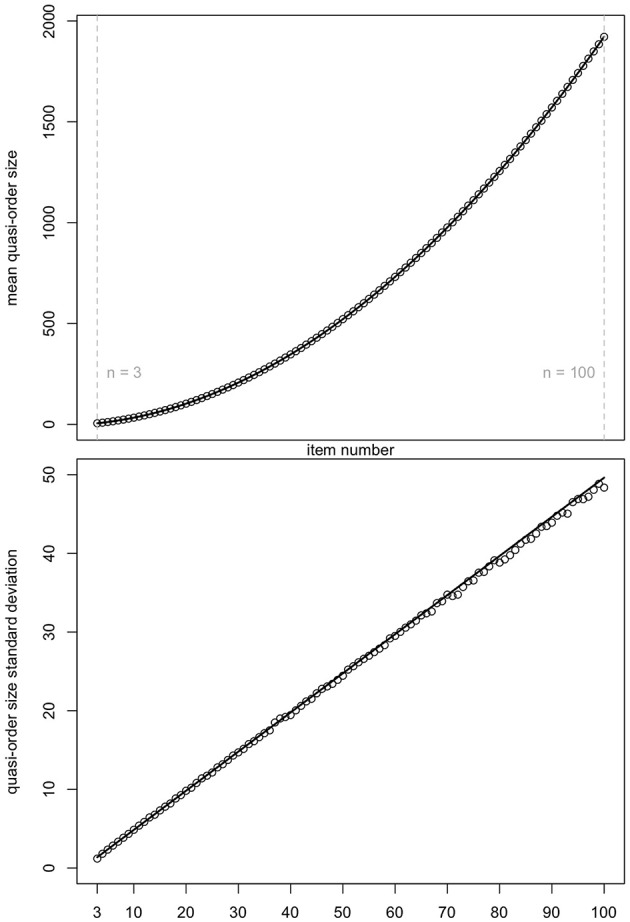
For item numbers *n* = 3, …, 100, the predicted by regression (solid lines) and the observed in NLSM samples of size *N* = 10, 000 quasi-orders (unfilled circles) means and standard deviations of the quasi-order sizes are compared, in top and bottom panels, respectively.

In Figure 12, for *n* = 3, …, 100, the means and standard deviations of the quasi-order sizes computed in NLSM samples of size *N* = 10, 000 quasi-orders (unfilled circles) are compared with the values predicted by regression (solid lines).

In Figure 12, we see a good agreement of the regression predicted and NLSM observed values for the item numbers *n* = 3, …, 100. In particular, the mean quasi-order size is more stable than the quasi-order size standard deviation.

**Figure 13:**
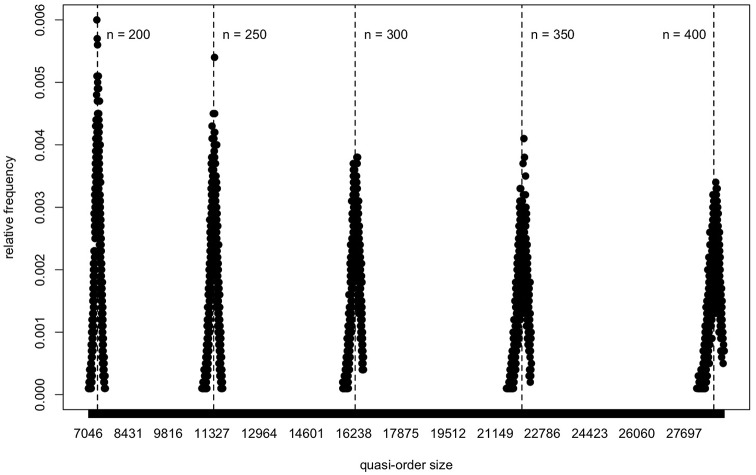
For the NLSM method, the quasi-order size distributions are portrayed for the large item numbers *n* = 200, 250, 300, 350, and 400. One sample of *N* = 10, 000 quasi-orders is simulated for any *n*. The five distributions are plotted using a common scale for the *x*-axis. The vertical dashed lines give means of the quasi-order sizes computed in the samples.

In Figure 13, based on the NLSM method, we plot the quasi-order size distributions obtained in samples of size *N* = 10, 000 quasi-orders, for the item numbers *n* = 200, 250, 300, 350, and 400. For these plots, location-scale matching used for bias correction was applied successively in each individual inductive step of the RDIC procedure. At this point, a remark is in order. For larger *n*, approximately *n*≥150, the RDIC samples have only a small overlap with or are separated and disjoint from the NLSM and DLSM support ranges. This artifact can also occur with other (e.g., *ad hoc*) sampling procedures (section 2), if we use these in lieu of the RDIC in the formulation of the NLSM or DLSM routines (cf. also last paragraph of section 6). Thus, there are a limited amount of or no quasi-orders available that could be resampled to become the NLSM or DLSM graphs. A solution to this problem is to apply location-scale matching successively in each inductive step of the RDIC procedure. In contrast to other (e.g., *ad hoc*) sampling procedures, this is feasible with the RDIC routine because of its inductive setup. This was done for Figure 13.

In Figure 13, we can see, yet more clearly if we zoom in into the separate plots, that the distributions of the quasi-order sizes are roughly bell-shaped. A trend can be seen in Figure 13, in which all five distributions are plotted together using a common scale for the *x*-axis.

## 6. Conclusion

We summarize our findings and end with further research directions.

### 6.1. Summary

This work is a third paper of a series of articles contributing to the issue of representatively sampling quasi-orders. The two prior publications on this issue are Schrepp and Ünlü (2015) and Ünlü and Schrepp (2016b). In Schrepp and Ünlü (2015), the uniform extension method (UEM) was proposed. In Ünlü and Schrepp (2016b), the randomized doubly inductive construction (RDIC), simple resampling method (SIRM), and stratified resampling method (STRM) were introduced. In the present paper, we have described two further alternatives, the normal location-scale matching (NLSM) and discrete location-scale matching (DLSM) methods.

The UEM is the exact method, theoretically representative, but only works for small item numbers, up to *n* = 15. The SIRM and STRM methods, as bias correcting resampling strategies on the RDIC procedure, are approximate and provide close to representative quasi-order samples, computationally viable up to *n* = 50. The NLSM and DLSM techniques, on the other hand, significantly improve on the efficiency and feasibility of the afore mentioned methods. The NLSM and DLSM are only approximate methods, which we have demonstrated for item numbers up to *n* = 400.

To sum up, we have addressed why ordered structures including the quasi-orders are important, why we want to sample random quasi-orders representatively, and the broader scope of this paper (section 1). We have reviewed the currently available sampling techniques for quasi-orders, especially the UEM, RDIC, SIRM, and STRM methods (section 2). We have performed polynomial regression analyses for the mean quasi-order size and quasi-order size standard deviation as a function of item number (section 3). For the mean and standard deviation, we have seen that quadratic and linear relationships, that is, *q*(*k*) = −1.116+1.673*k*+0.176*k*^2^ and *l*(*k*) = −0.121+0.497*k*, respectively, do hold, with *k* the item number. We have introduced the new methods NLSM and DLSM (section 4). If *f*_μ, σ_ denotes the normal probability density function with mean μ and standard deviation σ, the defining probability weights of the NLSM approach are given by $f_{\mu =q\left(k\right),\sigma =l\left(k\right)}\left(s\right)/\underset{s^{\prime }\in S}{\sum }f_{\mu =q\left(k\right),\sigma =l\left(k\right)}\left(s^{\prime }\right)$. On the other hand, the DLSM method crucially rests on the transformed values $t\left(s\right):=\sigma_{t}\left(s-\bar{\mu }\right)/\bar{\sigma }+\mu_{t}$ (for notation details, see section 4). In simulations, the scope and usefulness of the methods NLSM and DLSM have been investigated (section 5). We have seen that the NLSM is the better performing method as compared to the DLSM. Forming their “Average” has slightly improved on the methods, for smaller item numbers. Overall, both the methods NLSM and DLSM have provided good approximations to representative reference values, with respect to criteria other than the size, but primarily in regard to representative quasi-order size distributions. The results obtained for the location parameter have been more robust than for the scale parameter. We have simulated large quasi-orders on up to *n* = 400 items and have observed roughly bell-shaped size distribution graphs.

### 6.2. Further Research

We conclude with suggestions for further research. A possible direction for future research may be the unlabeled, or isomorphic, sampling of quasi-orders. In this paper, only labeled quasi-orders have been considered. This would necessitate the development of some analog of the RDIC procedure, for the combinatorial construction of the representatives of all isomorphism classes and its proper randomization. Furthermore, generating combinatorial structures uniformly at random according to such procedures as the NLSM and DLSM could also be studied for ordered structures other than the quasi-orders. Examples are weak, partial, or linear orders. Literature such as Harary and Palmer (1973), Dixon and Wilf (1983), Kerber et al. (1990), Brinkmann and McKay (2002, 2005), Pfeiffer (2004), and Roberts and Tesman (2009) in mathematics, and Flajolet et al. (1994), Rodionov and Choo (2003), Duchon et al. (2004), and Bassino and Nicaud (2007) in computer science may prove valuable for these future research endeavors (section 1.3), albeit these works may not be directly applied in the present context of sampling quasi-orders (Ünlü and Schrepp, 2017).

Another interesting direction for further research may be the comparison of other methods with the RDIC procedure used to construct the quasi-orders underlying the NLSM and DLSM. In their current formulations, the NLSM and DLSM have at the basis the RDIC generated quasi-order samples (section 4). For this purpose, for instance the very flexible normal *ad hoc* sampling procedure or a very efficient variant of the entry-wise uniform sampling approach followed by taking the transitive closure (section 2) could be applied, to build the underlying quasi-orders that are being resampled according to the NLSM or DLSM procedures. Then, it remains to be seen how representative such modified NLSM and DLSM samples still are, if samples (for large *n*) can be obtained at all (penultimate paragraph of section 5).

## Author Contributions

AÜ conceived the matching methods. AÜ and MS designed the software used in analysis. AÜ and MS wrote the paper. All authors reviewed the manuscript, approving the final version of the paper prior to submission.

### Conflict of Interest Statement

MS was employed by company SAP SE. All other authors declare no competing interests.
